# Alterations in brain structure–function coupling associated with musical training

**DOI:** 10.3389/fnhum.2026.1873065

**Published:** 2026-07-07

**Authors:** Wenjie Li, Jing Luo, Xue Zhong, Daoqun Ding

**Affiliations:** 1 School of Educational Science, Cognition and Human Behavior Key Laboratory of Hunan Province, Institute of Interdisciplinary Studies, Hunan Normal University, Changsha, China; 2 Shaoyang Industry Polytechnic College, Shaoyang, China

**Keywords:** brain connectivity, MRI, music training, neuroplasticity, structural decoupling index

## Abstract

**Objective:**

Musical activity requires the coordinated integration of auditory, motor, and cognitive systems, making it an effective framework for studying brain plasticity. Previous studies have explored music training-related alterations in brain structure and function. However, the relationship between structure and function remains insufficiently understood. In this study, we applied a novel morphometric measure, morphometric inverse divergence, to assess the structural decoupling index (SDI) and evaluate differences in structure–function coupling between musicians and non-musicians.

**Methods:**

A total of 39 participants (22 musicians, 17 non-musicians) were enrolled. Group differences in SDI were examined to assess regional structure–function dependency. Within the musician group, correlation analyses were conducted between SDI and musical training experience and age of onset of training.

**Results:**

Compared to non-musicians, musicians exhibited significantly lower SDI in the left superior frontal gyrus, right inferior frontal gyrus, right superior temporal gyrus, right parahippocampal gyrus and left posterior superior temporal sulcus (pSTS). Notably, the SDI in left pSTS showed a significant negative correlation with cumulative musical training experience and a positive correlation with age of onset of training.

**Conclusion:**

These findings demonstrate that long-term music training is associated with enhanced structure–function coupling in fronto-temporal networks, particularly in regions supporting auditory-motor integration and memory. Furthermore, the left pSTS appears specifically sensitive to training intensity and timing.

## 1 Introduction

As a complex multisensory activity, music depends on a coupling of perception and action. This interaction engages sensory, motor, and multimodal integration regions and requires coordinated involvement of auditory, motor, and cognitive systems (MacLean et al., 2025). Such sustained engagement drives structural and functional brain reorganization, affecting perceptual, cognitive, and motor performance (Wan and Schlaug, 2010). Consequently, long-term music training has been shown to shape the structural and functional characteristics of the brain (Hyde et al., 2009; Kunikullaya et al., 2025). Beyond auditory-motor regions, musical training also engages higher-order cognitive and memory-related networks, including the prefrontal cortex and medial temporal regions (Bermudez et al., 2009; Fauvel et al., 2014).

Relative to non-musicians, musicians often exhibit increased gray matter volume in regions involved in auditory processing, motor control and emotional regulation, such as superior temporal gyrus (STG) and inferior frontal gyrus (IFG) (Liu et al., 2025). While these structural differences have been consistently replicated across studies, findings on functional activation patterns are less consistent. For example, some studies reported enhanced auditory-evoked responses in musicians (Musso et al., 2020), whereas others have no significant group differences (Olszewska et al., 2021). Such discrepancies suggest that the relationship between brain structure and function may not be simply linear, highlighting the need for more integrated analytical approaches.

There is growing recognition that the brain operates as an integrated system wherestructure and function are dynamically interdependent (Bullmore and Sporns, 2009). Accordingly, studies have increasingly focused on the integrated analysis of structure and function to better understand brain organization (Xiayan et al., 2025). Earlier studies investigated structure and function coupling through quantified linear correlations between structural and functional connectivity (Honey et al., 2009). However, accumulating evidence indicates that the function and structure coupling exhibits a complex phenotype that linear methods may not fully capture (Medaglia et al., 2018; Sun et al., 2024).

To overcome this limitation, the structural decoupling index (SDI) has emerged as a novel metric to quantify the degree of structure–function decoupling across brain regions. Unlike global correlation measures, SDI reflects how much regional neural activity deviates from the underlying structural connectivity architecture (Preti and Van De Ville, 2019). This index has been applied to investigate brain characteristics in aging, neurodegenerative diseases and psychiatric disorders (Sun et al., 2024; Xiayan et al., 2025), as well as in healthy population such as pilots (Chen et al., 2025). Moreover, SDI has been shown to been sensitive to individual differences in cognitive performance and learning-related plasticity. As SDI captures region-specific deviations of function from structure, it is particularly well-suited for studying experience-dependent plasticity. Musical training engages distributed auditory-motor, cognitive, and limbic networks and may induce heterogeneous decoupling patterns across regions—some areas becoming more tightly coupled and others more flexibly decoupled as a result of training. However, how musical training relates to brain structure–function coupling at the regional level levels remains largely unclear.

Beyond the general effects of training, the age of onset of training and the cumulative amount of musical experience have been identified as an important modulator of training induced neural plasticity (Merrett et al., 2013). Specifically, early initiation of training during developmental sensitive periods has been associated with greater structural and functional specialization, particularly in auditory-motor networks (Penhune, 2011). Likewise, greater cumulative practice has been linked to more pronounced neuroplastic changes, including increased gray matter volume and enhanced functional connectivity (Gaser and Schlaug, 2003; Palomar-García et al., 2020). Collectively, these factors may associate with training-associated brain plasticity, yet the association between neuroplastic alterations and individual training parameters remains unclear.

To address these gaps, the present study adopted the SDI framework to explore alterations in brain structure–function decoupling patterns associated with musical training. Based on previous evidence, we hypothesized that musicians would exhibit lower SDI than non-musicians within brain networks critical for auditory-motor integration, cognitive control and musical memory. These networks include temporal regions supporting auditory perception and sensorimotor mapping, frontal regions involved in executive functions and attentional control and medial temporal regions associated with musical memory. Furthermore, we explored whether individual training parameters-cumulative years of training and age of onset-correlated with SDI in regions showing group differences.

## 2 Materials and methods

### 2.1 Participants

A total of 39 participants were included in this study: 22 expert musicians, and 17 non-musicians (Table 1). We assessed the age of training beginning and training years in music group. The control group had no formal training in music. Inclusion criteria for musicians were: (i) ≥ 7 years of formal musical training; (ii) current active engagement in musical performance. Inclusion criteria for non-musicians were: (i) no formal musical training; (ii) less than 1 year of informal musical experience. The groups did not differ in age or sex distribution. All participants were right-handed. The study received approval from the Ethics Committee of local university of China. All participants provided written informed consent. This study adhered to the ethical principles outlined in the 1964 Declaration of Helsinki.

**Table 1:** Demographic characteristic of the participants.

Characteristic	Musicians	Non-musicians	*p*-value
Gender (F/M)	20/2	15/2	0.78^a^
Age	19.95 ± 0.95	20.53 ± 1.07	0.09^b^
Music-experience	10.27 ± 0.68	–	–
Age of music-training onset	9.05 ± 0.71	–	–

a *p* was calculated using the chi-square test.

b *p* was calculated using the two independent sample t-test.

### 2.2 Images acquisition and preprocessing

Imaging data were acquired on a 3.0-T Siemens MRI scanner. Resting-state functional MRI data were acquired using the following parameters: repetition time (TR) = 2000 ms; echo time (TE) = 30 ms; flip angle (FA) = 9°; field of view (FOV) = 220 × 220 m^2^; matrix = 64 × 64; number of slices = 32; slice thickness = 3 mm. T1-weighted anatomical images were collected using the following parameters: TR = 2,600 ms; TE = 3.02 ms; FA = 8°; FOV = 220 × 220 m^2^; number of slices = 176. We excluded those subjects with large head motion in any direction corresponding to >1.5 mm or any rotation >1.5°.

Functional images were preprocessed by DPABI,^1^ which included the following steps: (1) the first five time points of the time series were deleted for signal equilibrium and allow the participants to adapt the scanning noise; (2) slice timing; (3) realigning; (4) normalization, functional images were coregistered with corresponding T1-weighted structural images, normalized to the Montreal Neurological Institute space (voxel size = 3 mm × 3 mm × 3 mm); (5) detrending; (6) regressing nuisance signals, including the Friston-24 motion parameter, white matter signal, cerebrospinal fluid signal; (7) temporal filtering was performed at bandpass 0.01–0.08 Hz. No spatial smoothing was applied during preprocessing, as it would blur functional boundaries across adjacent regions and bias decoupling estimates toward homogeneity (Preti and Van De Ville, 2019).

For each participant, the T1-weighted images were preprocessed using the recon-all pipeline from FreeSurfer software version 7.4.1^2^ within a surface-based framework. Structural preprocessing included skull removal, tissue’s segmentation, segmentation of hemispheric and subcortical signatures, and reconstruction of gray-white matter boundaries and cortical surfaces.

### 2.3 Construction of morphometric inverse divergence network

Cortical surface reconstruction and feature extraction were performed using FreeSurfer’s recon-all pipeline (Fischl, 2012). For each participant, five morphological features (gray matter volume, surface area, cortical thickness, sulcal depth and mean curvature) were extracted (Sebenius et al., 2023). Cortical parcellation was performed using the Brainnetome Atlas (BN Atlas) (Fan et al., 2016), which provides 210 cortical regions. Morphometric Inverse Divergence (MIND) networks were constructed by calculating the symmetric Kullback–Leibler divergence between the multivariate feature distributions of each pair of cortical regions (Sebenius et al., 2023). This yielded a 210 × 210 MIND network matrix for each participant, which served as the basis for subsequent analyses. After obtaining the 210 × 210 MIND matrix, Fisher’s z-transformation was applied to normalize the correlation distribution.

### 2.4 Measurement of structural decoupling index

**Figure 1:**
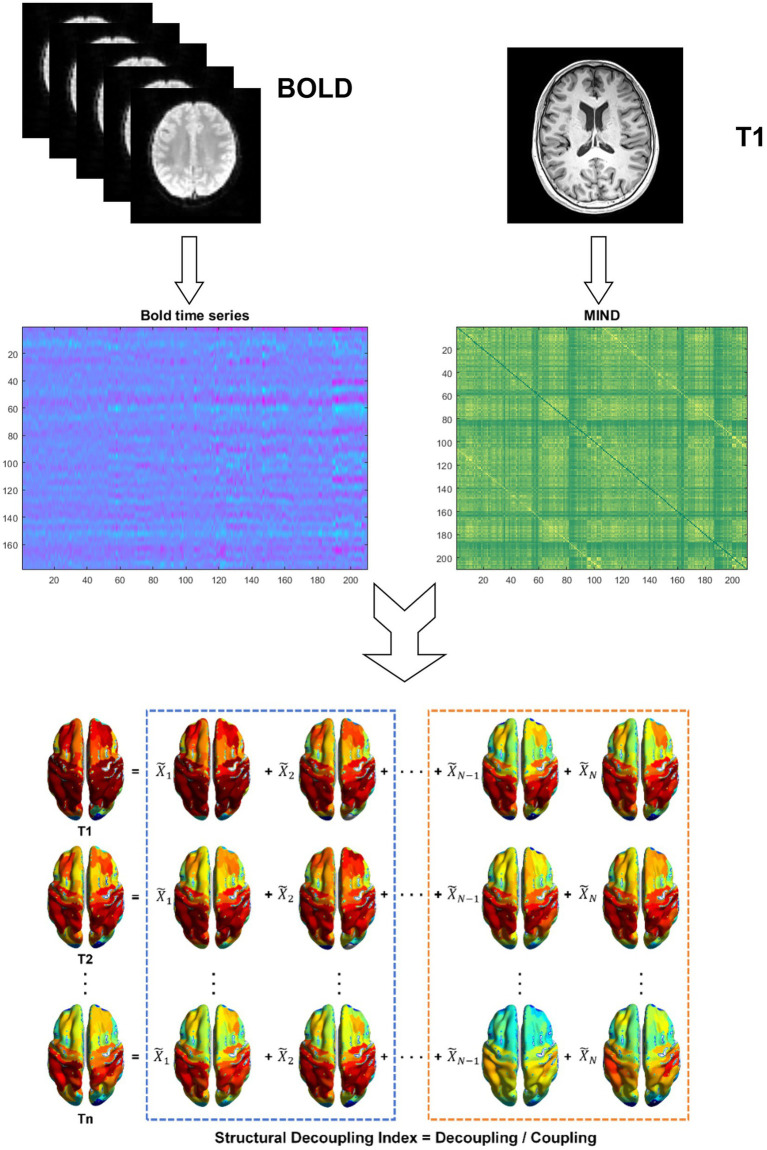
SDI construction.

For each participant, functional time series were extracted from the 210 cortical regions defined by the BN Atlas (Fan et al., 2016), using the same parcellation scheme employed for MIND network construction. This yielded an N × T time-series matrix per participant, where N = 210 (cortical regions) and T = 178 (time points). These functional time series were then combined with the participant-specific MIND structural network to compute the SDI at the regional level (Preti and Van De Ville, 2019). The SDI quantifies the degree of structure–function coupling, with lower values indicating stronger coupling and higher values indicating weaker coupling (Preti and Van De Ville, 2019). This procedure resulted in a 1 × 210 SDI vector per participant, representing regional SDI values for the 210 cortical regions, which were subsequently used for statistical analyses (Figure 1) (Dong et al., 2024).

### 2.5 Statistical analysis

Independent-sample *t*-tests were performed to compare SDI values between the musician and non-musician groups across 210 cortical regions, with Benjamini-Hochberg FDR correction (*p* < 0.05) applied at the regional level for multiple comparisons. For regions showing significant group differences, Pearson correlations between SDI values and years of formal training, as well as age of training onset in musician group. For these correlation analyses, FDR correction was applied across the two correlations, with statistical significance set at *p* < 0.05.

## 3 Results

### 3.1 Group difference in structural-functional coupling

**Figure 2:**
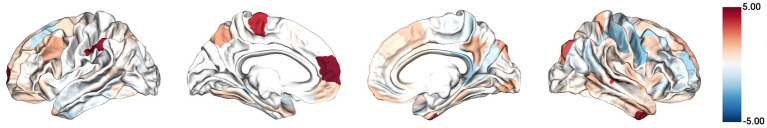
*t*-value maps of group differences in SDI between musician and non-musician groups. “Red indicates regions where non-musicians showed significantly higher SDI than musicians”.

**Figure 3:**
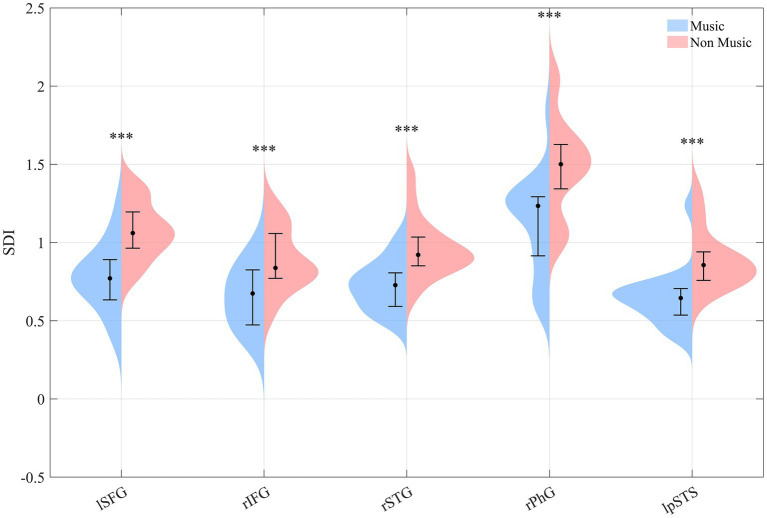
Comparison of SDI between musicians and non-musicians in significant brain regions. ***p < 0.001.

Compared with non-musicians, musicians showed significant lower SDI in the left superior frontal gyrus, right inferior frontal gyrus, right superior temporal gyrus, right parahippocampal gyrus and left posterior superior temporal sulcus (Table 2; Figures 2, 3).

**Table 2:** Brain regions showing significant SDI differences between musicians and non-musicians.

Brain regions	Hemisphere	*t*-value	*p*-value	Cohen’s d
SFG	Left	4.72	0.007	1.53
IFG	Right	3.89	0.021	1.26
STG	Right	4.45	0.008	1.43
PhG	Right	3.76	0.024	1.22
pSTS	Left	4.33	0.008	1.40

### 3.2 Relationships between SDI and music training experience

**Figure 4:**
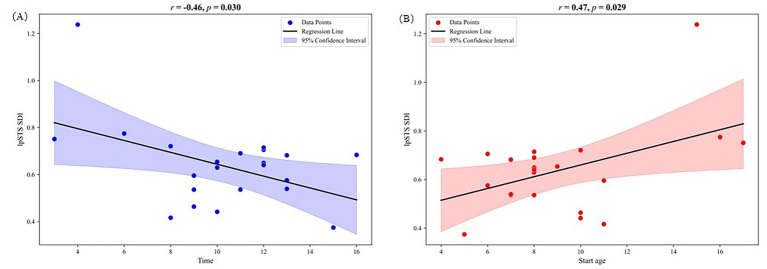
Correlation between SDI in lpSTS and music training experience. **(A)** “Time” refers to cumulative training experience. **(B)** “Start time” refers to age of training onset.

Within the musician group, SDI in pSTS exhibited significant negative correlations with music training time (r = −0.46, *p* = 0.030, Figure 4A). Additionally, a significant positive correlation between SDI in lpSTS and the start time was observed (*r* = 0.47, *p* = 0.029, Figure 4B). All reported *p*-values for correlations are FDR-corrected.

## 4 Discussion

In the current study, we investigated whether long-term musical training is associated with altered structural-function coupling using the SDI. Our findings support the hypothesis that musicians exhibit lower SDI than non-musicians, particularly in fronto-temporal regions supporting auditory-motor integration, cognitive control and musical memory. Furthermore, the correlations between SDI in left pSTS and training parameters (cumulative years and age of onset) suggest that intensity and timing of training are linked to the degree of coupling.

Musical performance places extraordinary demands on the integration of auditory perception and motor execution. The core auditory-motor integration network encompasses the posterior superior temporal sulcus and superior temporal gyrus, which serve as convergence regions for mapping auditory representations onto motor plans (Zatorre et al., 2007). We found reduced SDI in left pSTS and right STG in musicians, suggesting that musical training is associated with strengthened correspondence between structural connectivity and functional interactions in this network. Furthermore, the negative correlation between SDI in pSTS and cumulative musical experience indicates that more intensive training relates to more efficient structure–function coupling. These findings align with previous studies showing that music training is associated with structure–function coupling in sensorimotor networks, especially in regions involved in auditory-motor integration (Gao et al., 2024). Similarly, a recent review by Mucignat-Caretta and Soravia (2023) highlighted that musical training represents a positive environmental modulation that correlates with both structural and functional brain development. In line with this, early research demonstrated that structural alterations in auditory regions emerge relatively early during musical training, suggesting that the temporal cortex is highly responsive to musical experience (Groussard et al., 2014). Together, these results highlight that long-term musical engagement is associated with experience-dependent plasticity in the temporal cortex, promoting more efficient structural-functional coupling within the auditory-motor network. Such refined coupling may underlie the enhanced auditory-motor coordination and precise sensorimotor synchronization that characterize skilled musical performance. We note, however, that motor-related regions such as the precentral gyrus and supplementary motor area did not show significant SDI differences between groups in our study. Resting-state fMRI may not fully capture training-induced plasticity in motor regions that are primarily engaged during active performance. Future studies using task-based fMRI may help clarify the role of motor regions in music training-related plasticity.

The left superior frontal gyrus is critically implicated in executive functions such attention control, working memory and multi-task coordination (du Boisgueheneuc et al., 2006). Concurrently, the right inferior frontal gyrus has been identified as a key node for rhythm processing and phrase segmentation (Grahn and Rowe, 2009). Musical performance requires sustained attention and real-time coordination of multiple tasks. The reduced SDI observed in these frontal regions suggest that long-term musical training correlates with strengthened structure–function coupling in frontal regions, potentially supporting more efficient cognitive control during complex musical performance (Halwani et al., 2011). This pattern converges with recent evidence that musical training correlates with enhanced structural and functional plasticity in frontoparietal and prefrontal systems underlying attention and cognitive control (Manting et al., 2025). Moreover, a recent work confirms that musical training robustly improves executive functions by shaping structural and functional properties of the superior and inferior frontal gyri (Cai et al., 2025). Collectively, these findings indicate that musical experience is associated with adaptive plasticity in frontal structure–function relationships.

The right parahippocampal gyrus showed reduced SDI in musicians. The parahippocampal cortex is involved in episodic memory and the encoding of contextual information (Eichenbaum et al., 2007). This region plays a specific role in music-evoked memory retrieval. For instance, Lesiuk et al. (2025) found that listening to familiar music elicited greater activation in the right parahippocampal gyrus than unfamiliar music, suggesting its involvement in music-evoked autobiographical memory. Similarly, Bonetti et al. (2024) revealed that the hippocampus and parahippocampal cortex forms part of a network underlying the recognition of memorized musical sequences. Enhanced coupling in this region may reflect the heightened demands of musical memory, including the retrieval of familiar pieces, structural regularities and performance contexts (Herholz and Zatorre, 2012). Taken together, these findings indicate that long-term musical training is associated with strengthened structure–function coupling in the parahippocampal region, highlighting the role of this area in musical memory processes.

Among the five regions with significant group differences, only the left pSTS showed correlations with training parameters. This regional dissociation may reflect the pSTS, as a hub for auditory-motor integration, continuously refines its structure–function coupling with increasing training intensity and earlier onset (Penhune, 2011). In contrast, frontal (SFG, IFG) and parahippocampal regions may undergo rapid plasticity early in training, reaching a ceiling in expert musicians, such that individual differences in cumulative years no longer predict coupling strength. Alternatively, resting-state fMRI may be less sensitive to training-related variation in these regions. Future longitudinal studies are needed to test these possibilities.

Several limitations should be acknowledged. First, the sample size is modest, although it is comparable to previous neuroimaging studies on musical training (e.g., Hyde et al., 2009). Nevertheless, future studies with larger samples are needed to replicate and extend our results. Second, although musicians and non-musicians did not differ significantly in age, sex, education, or head motion, we did not include these variables as covariates in the primary statistical models. Future studies with larger samples should consider covarying these factors to provide even stricter control. Third, we did not formally assess congenital amusia, absolute pitch, or socioeconomic status. We recognize that these factors could be relevant to musical training research. However, congenital amusia is relatively rare (Pfeifer and Hamann, 2015), and absolute pitch is even rarer in the general population (Herceg and Szabó, 2023). Additionally, our participants were recruited from the same university with comparable educational backgrounds, suggesting relative SES homogeneity. Nevertheless, we acknowledge this as a limitation and hope future studies can incorporate formal assessments of these factors.

## 5 Conclusion

In summary, musical training is associated with enhanced structure–function coupling in fronto-temporal regions supporting auditory-motor integration, cognitive control, and memory. The left pSTS, correlated with both training intensity and onset age, appears particularly sensitive to training parameters. These findings demonstrate that experience-dependent plasticity involves tighter alignment between brain structure and function.

## Data Availability

The raw data supporting the conclusions of this article will be made available by the authors, without undue reservation.

## Footnotes

1
http://rfmri.org/dpabi

2
http://surfer.nmr.mgh.harvard.edu/
